# 

**Published:** 1889-02-23

**Authors:** 


**PERMANENTLY ENLARGED TO 32 PAGES.**

PRICE ONE PENNY.

No. 126, Vol. V.—February 23rd, 1889.

[Registered at the General Post Office as a Newspaper.]

Per Annum, 6s. 6d., post free.

Monthly Parts. 6d.; post free, 7½d.

Ditto per Ann. 7s. 6d., post free.

